# Infection with a Virulent Strain of *Wolbachia* Disrupts Genome Wide-Patterns of Cytosine Methylation in the Mosquito *Aedes aegypti*

**DOI:** 10.1371/journal.pone.0066482

**Published:** 2013-06-19

**Authors:** Yixin H. Ye, Megan Woolfit, Gavin A. Huttley, Edwige Rancès, Eric P. Caragata, Jean Popovici, Scott L. O'Neill, Elizabeth A. McGraw

**Affiliations:** 1 School of Biological Sciences, Monash University, Melbourne, Australia; 2 The John Curtin School of Medical Research, The Australian National University, Canberra, Australia; International Atomic Energy Agency, Austria

## Abstract

**Background:**

Cytosine methylation is one of several reversible epigenetic modifications of DNA that allow a greater flexibility in the relationship between genotype and phenotype. Methylation in the simplest models dampens gene expression by modifying regions of DNA critical for transcription factor binding. The capacity to methylate DNA is variable in the insects due to diverse histories of gene loss and duplication of DNA methylases. Mosquitoes like *Drosophila melanogaster* possess only a single methylase, DNMT2.

**Description:**

Here we characterise the methylome of the mosquito *Aedes aegypti* and examine its relationship to transcription and test the effects of infection with a virulent strain of the endosymbiont *Wolbachia* on the stability of methylation patterns.

**Conclusion:**

We see that methylation in the *A. aegypti* genome is associated with reduced transcription and is most common in the promoters of genes relating to regulation of transcription and metabolism. Similar gene classes are also methylated in aphids and honeybees, suggesting either conservation or convergence of methylation patterns. In addition to this evidence of evolutionary stability, we also show that infection with the virulent *w*MelPop *Wolbachia* strain induces additional methylation and demethylation events in the genome. While most of these changes seem random with respect to gene function and have no detected effect on transcription, there does appear to be enrichment of genes associated with membrane function. Given that *Wolbachia* lives within a membrane-bound vacuole of host origin and retains a large number of genes for transporting host amino acids, inorganic ions and ATP despite a severely reduced genome, these changes might represent an evolved strategy for manipulating the host environments for its own gain. Testing for a direct link between these methylation changes and expression, however, will require study across a broader range of developmental stages and tissues with methods that detect splice variants.

## Introduction

Methylation at the cytosine base, which is the most common form of DNA methylation, is present in all vertebrates and flowering plants, some fungi and protozoa and many bacterial species [1]. Methylation is just one of a suite of reversible epigenetic mechanisms including histone modification [2] and small interfering RNA (siRNA) and microRNA [3], that allow for plastic changes in phenotype without a change in genotype [4]. A clear picture of the mode of action of methylation at the molecular level is still emerging. The initial sense was that methylation regulates transcription [5] by silencing promoters [1]. In some taxa, methylation is associated with transposons and so could also play a role in suppressing their activity [6], [7]. More recently it has been shown that methylation is also involved in alternate splicing and hence intragenic sites [8], [9].

The primary agent of cytosine methylation is a family of enzymes called methyltransferases or DNMTs. Insect genomes are highly variable with respect to the presence of these genes due a complex history of both gene loss and duplication [10]. As each of these genes are thought to have different functional roles, insects also vary largely in their capacity for methylation [4]. *Drosophila melanogaster* for example lacks the enzymes responsible for maintenance (DNMT1) and *de novo* methylation (DNMT3) [11], but does have DNMT2. This enzyme appears to only methylate DNA randomly with respect to genomic region, with activity peaking in early embryos [12], [13], [14]. It has also been shown to methylate tRNAs [15], [16]. When DNMT2 is knocked out in the fly, there is no clear phenotypic effect [13], [15], [17] although overexpression of the enzyme lengthens fly lifespan [18]. The honeybee genome in contrast harbours all three methylase genes plus a duplicated copy of DNMT3 [10]. Methylation is common throughout the genome and is associated with differential gene expression in castes [8], [19]. Mosquitoes are more similar to *D. melanogaster* with only DNMT2 present in the genomes of *Anopheles gambiae* [20] and *Aedes aegypti* [21].

*Wolbachia pipientis* is a maternally inherited intracellular bacterium that is found in a wide range of arthropod species including ∼40% of all insect species [22]. *Wolbachia* induces diverse reproductive abnormalities in its hosts [23]. A virulent strain of *Wolbachia*, *w*MelPop, which was originally found to cause early death in *D. melanogaster* [24] was stably transinfected into *A. aegypti* and shown to approximately halve the lifespan of its host [25]. A reduced lifespan in mosquitoes could potentially limit transmission of viruses and parasites, as it is often only older mosquitoes that are capable of transmitting these agents because of the time it takes a parasite or virus to migrate from an infected blood meal in the gut to the insect's salivary glands [26], [27]. While the basis of the early death phenotype is not known, *w*MelPop differs from other *Wolbachia* strains in that it grows to high densities, particularly in the brain but also in a range of other somatic tissues both its native [24] and novel mosquito host [25], [28]. As mosquitoes carrying *w*MelPop age they begin to demonstrate a range of abnormalities including increased activity and locomotor defects [29], [30], [31].

Infections with bacterial pathogens have been shown to alter the epigenome of vertebrate hosts [32]. The best-described examples are *Helicobacter pylori* [33] and uropathogenic *Escherichia coli* [34] where alterations of methylation profiles are responsible for characteristic changes in host gene expression. *Wolbachia* infection has also been shown to alter its host epigenetic makeup in a number of recent studies. Histone deposition in the male pronucleus of *Wolbachia*-infected *Drosophila simulans* may explain the expression of the symbiont-induced reproductive manipulation, cytoplasmic incompatibility (CI) [35]. In *D. melanogaster*, *Wolbachia* infection reduces the expression of a histone chaperone gene (*Hira*) in a manner that correlates with CI expression [36]. In a leafhopper, methylation profiles correlate with the expression of another *Wolbachia*-induced trait, feminization [37]. Lastly, in *A. aegypti*, *w*MelPop infection induces the production of a miRNA that positively regulate a metalloprotease gene. Inhibition of the miRNA or silencing of the target gene led to reductions in *Wolbachia* density in host tissues [38].

Here, we aimed to describe the methylome of *A. aegypti* and determine if it is altered by infection with the virulent *w*MelPop *Wolbachia*. We found that ∼1000 genes in adult *A. aegypti* were methylated and that these genes were more likely to exhibit reduced transcription. These genes were enriched for particular biological functional roles that were common to the honeybee and aphid methylome as well. While these findings demonstrated a level of stability in methylation patterns, the *w*MelPop infection also revealed a capacity for plasticity, with the infection both leading to new methylation events and removal of old signals. Most of these changes appear to be random with respect to gene function as per enrichment analysis and not clearly linked to changes in expression of associated genes. We hypothesise that many of these changes may be non-adaptive and evidence of a “sick” host exhibiting dysregulation of transcription. The only functional category of genes exhibiting enrichment was the GO term membranes (0016020). These methylation changes, if targeted, may reveal virulence associated effects on hosts or more basal aspects of *Wolbachia*:host interactions common to all *Wolbachia*. Given that *Wolbachia* is surrounded by a membrane of host origin [39], manipulating host transport and communication across membranes may represent an evolutionary adaptation to its intracellular habitat.

## Methods

### Ethics statement

Approval for blood feeding by human volunteers for maintenance of the mosquito colony was granted by The University of Queensland Medical Research Ethics Committee (2007001379). Volunteers provided written informed consent to participate.

### Mosquito rearing

Two laboratory lines of *A. aegypti* were used throughout this study. The PGYP1 line was generated by transinfection with *w*MelPop and this line was treated with the antibiotic tetracycline and cured from *Wolbachia* infection to create the PGYP1.tet line [25]. Mosquitoes were reared under standard laboratory conditions (26±2°C, 12:12 light/dark cycle, 75% relative humidity). Mosquito larvae were fed 0.1 mg/larva of TetraMin Tropical Tablets once a day. Adults were transferred to cages (measuring 30×30×30 cm) at emergence at 400 individuals per cage. Adults were supplied with a basic diet of 10% sucrose solution.

### Methylation microarrays

#### Sample collection, preparations and hybridization

Adult female mosquitoes were collected at 15 days as populations at this point are beginning to experience life shortening [25]. Mosquitoes were collected in pools of 20 and extracted for DNA using DNeasy spin columns (QIAGEN, Australia). Genomic DNA (INPUT, labeled Cy5) was fragmented using restriction enzyme *MseI* (5′−T^▾^TAA). Methylated DNA (IP, labeled Cy3) was enriched using an anti 5-methyl cytidine antibody (Abcam plc, UK). IP and INPUT DNA were separately amplified using a whole genome amplification method with universal oligonucleotide primers (WGA2, Sigma Aldrich, Australia) and 4 µg of amplified DNA was sent to Roche NimbleGen Systems for hybridization.

#### DNA methylation array design, hybridization and analysis

The DNA methylation arrays were designed and constructed by Roche NimbleGen (Madison, WI, USA). Given the size, it was not possible to tile the entire genome on a single chip. The array consisted of 713,540 custom designed probes of 64–80 bp length that tile the coordinates for regions −1300 bp to +500 bp around the promoter of all 17,416 mRNAs of the *A. aegypti* genome (retrieved from BioMart in Vectorbase AaegL1.2). Each probe was designed to have a predicted melting temperature of around 76°C. Unique probes were selected whenever possible; repeated masking of up to 10 matches between a probe and the genome was allowed. Three replicate hybridisations were carried out for each mosquito line. Raw signal intensity data was extracted from the scanned images of each array using Roche NimbleGen NimbleScan software and returned for analysis. Each feature on the array had a corresponding scaled log_2_ ratio, which was the ratio of the input signals for the treatment and control samples. The log_2_ ratio was scaled to centre the around zero. From the normalized log_2_ ratio, a fixed-length window (750 bp) was placed around each consecutive probe and a one-sided Kolmogorov-Smirnov (KS) applied to determine if probes were drawn from a significantly more positive distribution of log_2_ ratios than those from the rest of the array. The resulting score for each probe is the −log_10_ p-value from the windowed KS test around that probe. Peaks with at least 2 probes above a minimum P-value cutoff (−log_10_) of 2 were used for subsequent analysis.

A custom Perl script was then used to identify peaks that were unique to each line. As there are variations between biological replicates of the same line and methylation peaks may start and end at different coordinates, a conservative approach of calling peaks was employed. For a peak to be called, all three replicates of the line needed to have exactly the same starting and ending coordinates for that peak, while no peak was detected in the same region for any of the replicates of the opposite mosquito line. Peaks that were found only in PGYP1 and not in PGYP1.tet were considered to be methylation additions made in the presence of *w*MelPop. Peaks that were present exclusively in PGYP1.tet line represent removal of methylation in the presence of *w*MelPop.

Functional annotations of *A. aegypti* genes were retrieved from Biomart [40] in Vectorbase [41] and analyzed using the Ontologizer software [42], [43]. As functional annotation of the *A. aegypti* genome is less complete than that of the *D. melanogaster* genome, we drew on the functional annotations of *D. melanogaster* orthologs of *A. aegypti* genes for some of the fine-grained downstream analyses of functional enrichment. *D. melanogaster* orthologs of *A. aegypti* genes were retrieved from BioMart in Vectorbase [40], [41]and further analyzed using the online Database for Annotation, Visualization, and Integrated Discovery (DAVID) [44], [45]. “Transposable element data was obtained from Ensembl genomes release 5. We used the Ensembl querying capabilities of PyCogent [46] to identify transposons located within the annotated coordinates of *Aedes aegypti* genes. Transposons whose repeat class was specified as “unknown” or “dust” were ignored.

### Gene expression microarrays

#### Experimental design

Whole-genome microarrays were used to compare gene expression of PGYP1 relative to PGYP1.tet in head and muscle tissues independently using a dual-color reference design. These two tissues were selected for analysis given the particular tropism of *w*MelPop for brain tissue [24] and because defects in these tissues could underpin the locomotory changes associated with *w*MelPop [29], [30], [31]. Each tissue type was represented by technical replicates (N = 3) that were then replicated with a dye swap (total N = 6). Microarrays were of the 4×44 K format (Agilent) each containing standard control features and 3 replicates of each 60 mer feature randomly distributed across the layout. The *A. aegypti* genomic sequence (Vectorbase genome build 1.1) was used for construction of oligonucleotide microarrays using eArray Version 5.0 (Agilent Technologies Inc., Santa Clara, CA). After removing probes that cross-hybridized, a total of 12,336 transcripts which represented 12,270 genes were spotted onto each microarray.

#### Sample collection

Head and muscle tissues were dissected from 15 day old females into PBS in pools of 20, snap frozen in liquid nitrogen and extracted for Total RNA using Trizol (Invitrogen Corp., Carlsbad, CA). RNA was then purified using RNeasy kits (Qiagen, Australia) according to the manufacturer's instructions. Further sample preparations and hybridizations were then carried out by the Special Research Centre Microarray Facility at the University of Queensland. Sample quality was examined using the Agilent 2100 Bioanalyzer (Agilent Technologies Inc., Santa Clara, CA). Fluorescent cDNA was synthesized using Agilent Low RNA Input Linear Amplification Kit with Cyanine 3 or Cyanine 5-CTP.

#### Data analysis

For each transcript, raw data was extracted and analyzed using Genesping v.9.0 (Agilent Technologies Inc., Santa Clara, CA). An intensity dependent (Lowess) normalization (Per Spot and Per Chip) was used to correct for non-linear rates of dye incorporation as well as irregularities in the relative fluorescence intensity between the dyes. Hybridizations from each tissue type were used as replicate data to test for significance of expression changes using the cross-gene error model. The occurrence of false positives was corrected using the QVALUE [47], [48]. All array data have been deposited in ArrayExpress (http://www.ebi.ac.uk/microarray-as/ae/) under the accession E-MEXP-2907.

## Results

### Methylation in the*A. aegypti* genome

First, we characterised the basic patterns of methylation across the genome of *A. aegypti* not infected with *Wolbachia.* In brief, 1007 or 6% of the 16,340 genes represented on the tiling arrays showed evidence of methylation (Table S1), either in the promoter as defined as 1300 bp upstream from start (1.7%), the first 500 bp of the gene (2.2%) or in both regions (2.1%). In 75% of cases where there was methylation in any of these regions, at least one transposon was present in the gene compared with only 54% of cases when methylation was not present. Methylation was also more likely to be associated with genes of longer transcript length (Figure S1). The positive association between methylation and transcript length cannot, however, be explained simply by a greater likelihood of more CpG sites in the sequence, as transcript length in *A. aegypti* is negatively correlated with GC content of the gene (Figure S2). A test for enrichment by biological function was carried out in Ontologizer 2.0 for the methylated genes with all known *A. aegypti* genes as a population dataset [42], [43]. As the methylation tiling array contained probes for all known genes this is appropriate. Many of the significant GO terms were associated with ‘regulation’ (modulating the frequency, rate or extent of other processes) and in particular regulation of processes relating to transcription and metabolism (Table 1). Many of these functional classes are also over represented in the methylome of the pea aphid *Acythosiphon pisum* and the honeybee *Apis mellifera* [49] (Table 1).

**Table 1:** Biological process GO terms enriched among methylated genes in*A. aegypti*.

GO ID	Name	*P -value	Top 10 GO terms
GO:0050789	regulation of biological process	0.001	
GO:0065007	biological regulation	0.001	
GO:0006350	transcription	0.001	
GO:0019222	regulation of metabolic process	0.001	
GO:0006139	nucleobase, nucleoside, nucleotide and nucleic acid metabolic process	0.002	*A. pisum*, *A mellifera*
GO:0006807	nitrogen compound metabolic process	0.002	
GO:0009987	cellular process	0.004	*A. pisum*, *A mellifera*
GO:2000112	regulation of cellular macromolecule biosynthetic process	0.010	
GO:0031424	Keratinization	0.010	
GO:0010556	regulation of macromolecule biosynthetic process	0.012	
GO:0080090	regulation of primary metabolic process	0.013	
GO:0031323	regulation of cellular metabolic process	0.018	
GO:0031326	regulation of cellular biosynthetic process	0.018	
GO:0060255	regulation of macromolecule metabolic process	0.021	
GO:0044260	cellular macromolecule metabolic process	0.022	*A. pisum*
GO:0044237	cellular metabolic process	0.024	*A. pisum*, *A mellifera*
GO:0006464	protein modification process	0.027	
GO:0032774	RNA biosynthetic process	0.036	
GO:0010468	regulation of gene expression	0.036	
GO:0009889	regulation of biosynthetic process	0.039	
GO:0050794	regulation of cellular process	0.043	
GO:0030855	epithelial cell differentiation	0.046	

* Benjamini-Hochberg adjusted. Comparison with top 10 enriched categories of methylated genes of *A. pisum* [49] and *A. mellifera* [19].

**Figure 1:**
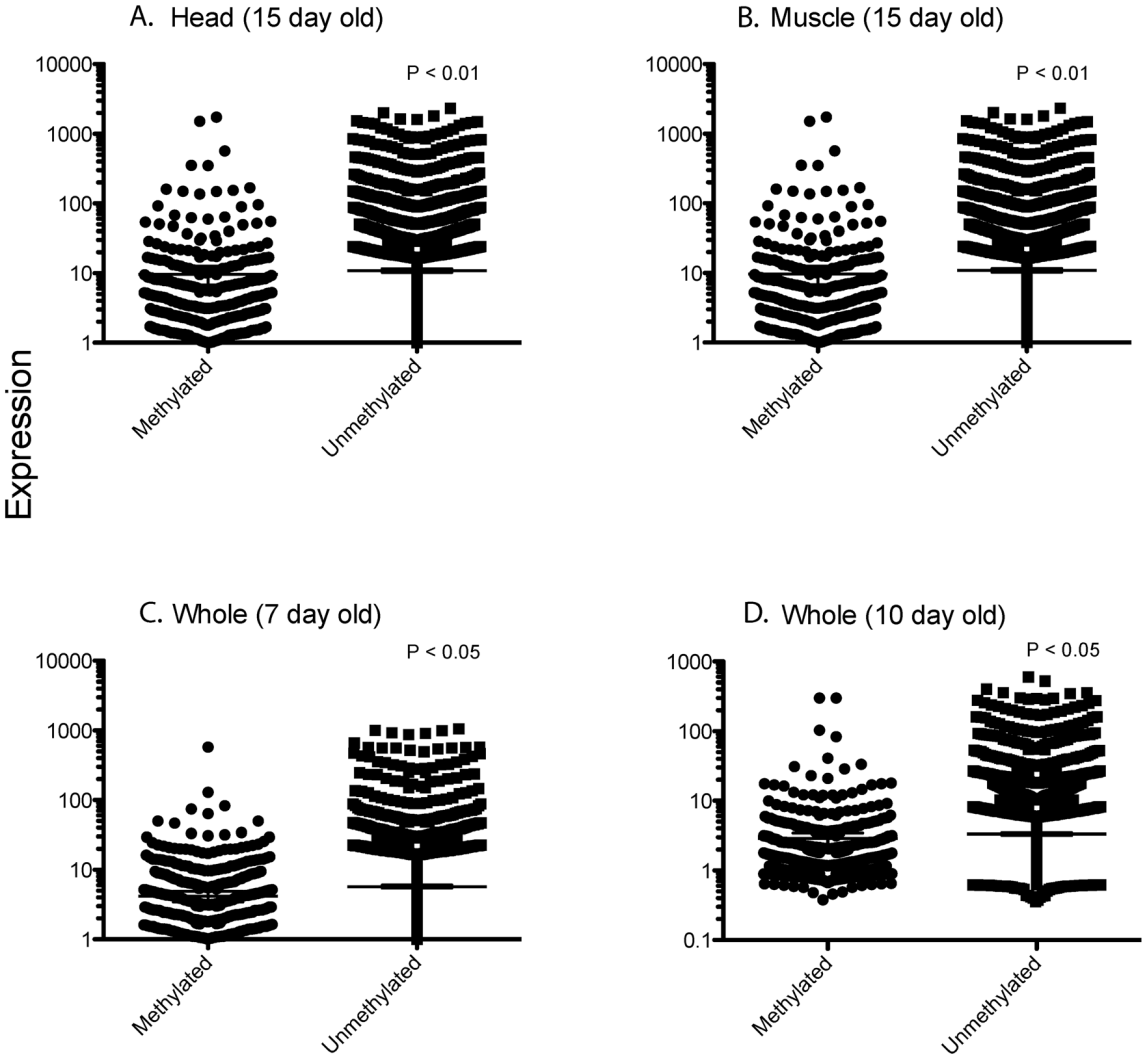
Gene regions with methylation are associated with lower levels of gene expression. Gene expression level is measured by fluorescence in single colour arrays for genes that are naturally methylated or unmethylated in *A. aegypti*. A. Head tissue, this study. B. Muscle tissue, this study. C. [51]. D. [50]. Mann-Whitney U-test, n in parentheses.

**Figure 2:**
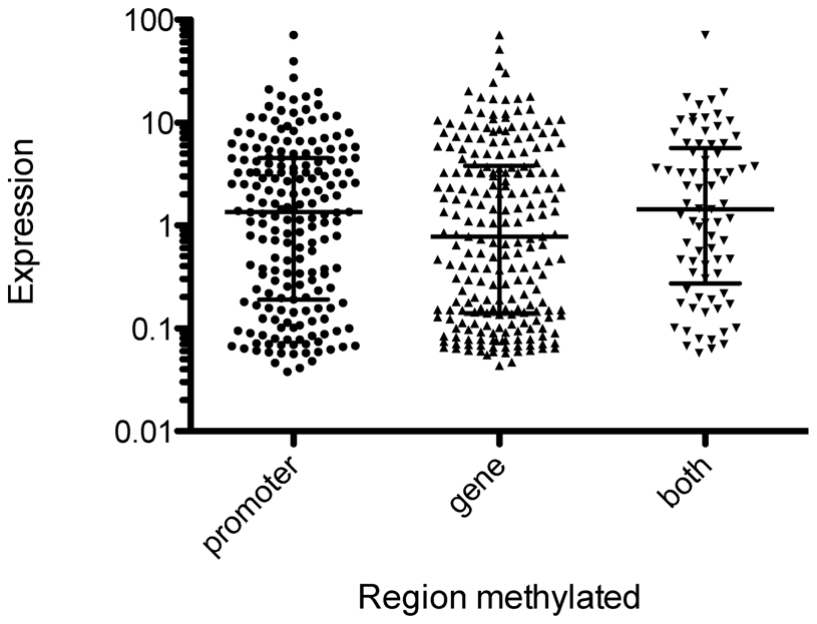
No difference in transcription levels for genes with methylation of the promoter versus the gene. Expression as measured by fluorescence in single colour arrays for methylated genes where methylation occurs at the promoter, within the gene, or both.

We then examined the transcriptional profiles of *A. aegypti* (not infected by *Wolbachia*) using single colour fluorescence data (Cy3) extracted from a range of dual colour experiments on whole mosquitoes [50], [51] and on specific mosquito tissues (this study). Gene regions with methylation were associated with lower levels of gene expression across all datasets (Figure 1), including profiles for adult mosquitoes aged ∼7 and ∼10 days [50], [51] and for head and muscle tissue taken from 15 day old adults (this study). When transcription levels were compared for genes with methylation of the promoter versus the gene (Figure 2), there was no significant difference (t = 1.25, df = 1, P = 0.21).

### *Wolbachia*'*s* effect on host methylation patterns

The presence of *w*MelPop infection led to the additional methylation of 63 genes in *A. aegypti* (Table S2). Only 40 of these genes were functionally annotated in *A. aegypti*, however, providing little statistical power for enrichment analysis. Nonetheless, these genes were compared in the Ontologizer to all annotated *A. aegypti* genes not already methylated [42], [43]. The top functional categories were membranes (GO:0016020) and calcium ion transmembrane transport (GO:0070588) both with p = 0.069 just beyond significance following multiple test correction. The presence of *w*MelPop infection was much more likely to be associated with demethylation events in the mosquito genome, affecting 699 genes or associated promoters in total (Table S3). The distribution of methylation loss events was similar between the regions examined: promoters (269), genes (222) or in both simultaneously (208). Of the list of demethylated genes, 347 were functionally annotated in *A. aegypti*. To test whether *w*MelPop-associated demethylation was targeted to genes involved with particular functions we identified enriched categories of biological function relative to all annotated genes that were already naturally methylated (536)[42], [43]. Only one GO term, membranes (GO:0016020), was significantly enriched after multiple test correction (p = 0.044). None of the genes assigned to this GO term that were only methylated or demethylated in response to *w*MelPop showed infection-associated changes in gene expression (Table S4) [51].

**Figure 3:**
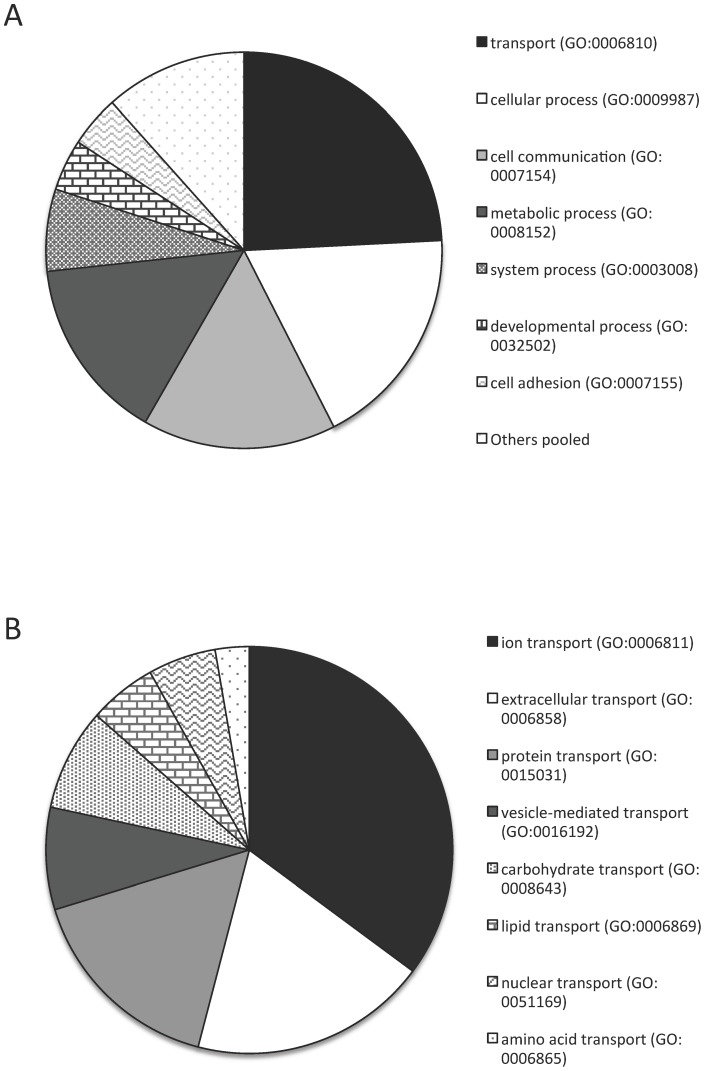
Pie chart of offspring categories comprising the GO group of membrane GO:0016020 (A). Pie chart of a further breakdown of the GO group of transport GO:0006810 (B).

To better understand the functional roles of genes ascribed to the GO term category, membranes (GO:0016020) we employed piechart analysis in Panther [52], [53] for all genes affected (either methylated or demethylated) by *Wolbachia* as a group (Figure 3A). For any GO term (the parent), this analysis reveals the genes representing each of the offspring GO terms or categories that comprise the parent. Of the 56 total genes in our list representing membranes (GO:0016020), the vast majority (29) were more specifically associated with the subcategory of membrane transport (GO:0006810). These 29 genes were mostly associated with only three offspring categories out of a total of 40 possible including; ion (GO:0006811), extracellular (GO:0006858) and protein (GO:015031) transport (Figure 3B). In addition to cellular transport, a large proportion of genes (22) were associated with the category of cellular processes (GO:0009987), comprised almost entirely by genes relating to cell communication (GO:0007154) (Figure 3A).

Lastly, we also examined the functional annotations for both the *A. aegypti* genes that undergo *w*MelPop-associated methylation changes and their *D. melanogaster* homologs to identify candidate methylation events that might underpin *w*MelPop-associated phenotypes in insects, including life shortening [24], [25] and defects in locomotory behaviours [29], [30], [31]. In the methylated category, genes of note included odorant receptor genes (AAEL000391, AAEL015506, AAEL011482), ion transporter genes with known effects on muscle contraction, locomotion and heart rate (AAEL013184, AAEL003837, AAEL005014) and genes involved with apoptotic cell clearance or regulation of apoptosis (AAEL012967, AAEL012970, AAEL011751). In the demethylated list, similarly there were genes associated with apoptosis (AAEL006633, AEL003444, AAEL001471), defense response (AAEL014744, AAEL007748, AAEL001932), phagocytosis (AAEL009732, AAEL012836, AAEL005378, AAEL008611), circadian rhythms (AAEL008141, AAEL006411, AAEL012960), lifespan (AAEL011465) and locomotion/flight behaviour (AAEL003608, AAEL014637, AAEL000466, AAEL001457, AAEL005302, AAEL012062, AAEL004717, AAEL006062, AAEL002522). When the transcriptional profiles were examined for these genes for muscle and head tissue (Table S4) or the whole insect [51] expression ratios (*Wolbachia* infected/uninfected) were not significantly different from one.

## Discussion

We have revealed low-level cytosine methylation throughout the *A. aegypti* genome both in promoters and within genes. Enrichment analysis has indicated that genes exhibiting methylation may be non-random with respect to biological function, with an over representation of regulators of transcription and metabolism. These findings are in contrast to a previous study in *Drosophila melanogaster*, another DNMT2-only organism, which suggested that methylation may be random in the genome [12], [13]. The two studies differ substantially in their methods, however – genome wide tiling versus random bisulfite sequencing – making direct comparisons difficult. The similarity in the types of genes likely to be methylated across bees, aphids [49] and *A. aegypti* suggests there may be inheritance of ancestral methylation states and/or strong selection pressure acting on methylation signals. Detailed evolutionary analysis of particular genes would be required to tease apart the relative contributions of these forces. Methylation patterns also change in response to *w*MelPop infection indicating some potential for plasticity, even in the adult stage. It remains to be seen whether the methylation/demethylation activity documented here is due to the action of DNMT2 or another yet-to-be discovered mechanism for methylation in this insect. The *w*MelPop-associated changes in *A. aegypti* methylation are not likely due to the direct action of *Wolbachia* methylases on host DNA. While experimental verification is needed, the *Wolbachia* genome contains 2 methylases associated with prophage regions whose annotation suggests they target adenosine not cytosine bases [54].

Environmental factors, including dietary manipulation are known to induce epigenetic changes in animals [55], [56]. A recent study in a pseudoscorpian, *Cordylochernes scorpioides* has shown that tetracycline alters sperm viability in a fashion consistent with a heritable epigenetic mechanism [57]. Tetracycline treatment is a commonly used approach to create *Wolbachia* cured lines with minimal genetic differences from infected lines, particularly following transinfection and artificial selection. Further study is needed to determine if CpG methylation is modified by tetracycline and if so whether the phenomenon is genome wide and generalizable to diverse taxa. If so tetracycline cured lines may differ from infected lines and in cases where the cured line has subsequently been introgressed into the *Wolbachia* infected line, methylome patterns will be manipulated.

The transcription of *A. aegypti* genes is lower where there is evidence of methylation, and this is independent of whether the methylation occurs in promoters or gene regions. On the whole, these data are in keeping with the idea that methylation is associated with transcriptional silencing [1]. The assumption of a clear relationship between CpG methylation and expression is likely overly simplistic, however, especially given that when we look at the expression of individual genes we do not see consistent patterns of methylation. Here, we have examined only a subset of *cis* sites, but other potentially relevant sites for transcriptional control are present further upstream of genes, inside exons or inside introns as is thought to be common in insects [4]. The expression of an individual gene may also be regulated in *trans* at remote sites for transcriptional regulators. Determining which sites matter will require tests of association with and deeper mapping of, sites either by complete genome tiling analysis or intensive gene-specific explorations. Also, if methylation regulates alternate splicing, the basic genome wide transcriptional profiling approaches like those employed here cannot detect and hence test for such differences [8], [9]. Lastly, transcriptional control may also be the result of multiple interacting epigenetic mechanisms including methylation, small or microRNAs [3] and histone modification [2].

This study demonstrates that infection with the virulent *w*MelPop *Wolbachia* strain is associated with widespread changes in host methylation. These changes for the most part do not appear to target particular classes of genes. This could be the result of low power with short gene lists, although it is worth pointing out that the increase in gene list size from ∼40 (methylated) to ∼350 (demethylated) did not bring about substantial increases in the number of significant gene categories. It is also possible that many of these genome-wide changes in methylation are not adaptive or specific manipulations by *Wolbachia*, but simply resulting changes in a sick and dying host. *w*MelPop-infected mosquitoes by 15 days of age are certainly exhibiting outward signs of their virulent infection including an innate immune response [51], [58], higher death rates [25] and defects in locomotor behaviours [29], [30], [31]. Evidence from other studies suggests that as animals age that their genome wide patterns of methylation become more variable possibly by loss of control of the processes that stabilize methylation [59], [60]. One explanation for *w*MelPop associated virulence traits is that the insects are simply experiencing accelerated aging. A parallel study in very old *A. aegypti* not infected with *Wolbachia* could reveal if there are similar changes in the methylome due entirely to age.

Interestingly, the lists of genes methylated and demethylated with *w*MelPop infection both showed the strongest enrichment of the same GO term: membranes. The effects of *w*MelPop on *A. aegypti* methylation are likely to include some changes that are the result of its unique virulence, particularly in a novel host [51], [61], as well as others that are common to all *Wolbachia* infections. Given the enrichment of membrane-associated genes, we hypothesize that these may represent the best candidates for fundamental host responses to *Wolbachia* infections. Inside insect cells, *Wolbachia* is housed within a membrane that is believed to be of host origin [39] and is the primary point of interaction between symbiont and host. The *Wolbachia* genome has lost many genes, in particular those involved in biosynthetic pathways, yet has retained a large number of transporters for amino acids, ATP and inorganic ions [62]. The host transport genes whose methylation was affected by *w*MelPop are mostly associated with inorganic ions, proteins and amino acids. After transport, *w*MelPop was most likely to affect genes in the cell communication category that was mainly comprised of signal transduction genes. If these methylation changes have phenotypic consequences, it is possible that *Wolbachia* has evolved a means to manipulate host cell transport and cellular communication to increase its access to nutrients or generally tailor the intracellular environment to its needs. *Wolbachia* possesses a functional type IV secretion system that could be used to directly influence host methylation [63]. In the first instance, the generality of the methyation changes in membrane-associated genes would need to be demonstrated in the methylome of other insects infected with non-virulent *Wolbachia* infections such *A. aegypti* and *D. melanogaster* infected with *w*Mel. If such changes were present, cell fractionation studies that partition *Wolbachia* and its vacuole from the rest of the host cell could be used to assess the distribution of ions, amino acids and proteins relative to uninfected cells. Lastly, the ability of *Wolbachia* to prevent a range of viruses, bacteria and parasites from replicating inside the mosquito cells it occupies has been recently demonstrated [28], [58]. Symbiont-associated changes in the membrane could provide mechanistic explanations for the exclusion.

## Conclusion

We have demonstrated the presence of methylation in the *A. aegypti* genome and that in general methylation status correlates with expression. We have identified similarities between *A. aegypti* and other insects in the functional classes of genes that are likely to be methylated that may suggest conservation or selection. At the same time, we also provide evidence that the methylome is plastic, changing in the face of infection with a virulent endosymbiont. These disturbances in patterns appear largely random with respect to gene function and do not affect transcription. The only functionally enriched class of genes is that of those involved in transport and communication across membranes which, given the nature of *Wolbachia*'*s* existence inside a host vacuole, may be a specific adaptation. The phenotypic consequences of these changes in methylation status are not clear and will require in-depth examination of gene expression across more developmental stages and between different tissues in response to diverse *Wolbachia* strains using methods that can detect alternatively spliced transcripts.

## Supporting Information

Figure S1 **Gene region with methylation are associated with greater transcript length as compared to gene regions without methylation.** Median transcript length (bp) ± interquartile range for genes whose promoters are naturally methylated or unmethylated in *A. aegypti*. *** P-value<0.001, n in parentheses.
(PDF)

Figure S2 **A negative correlation between GC% content of genes and gene length.**
(PDF)

Table S1 List of naturally methylated genes in *A. aegypti*. *A. aegypti* gene ID and description were compiled from Vectorbase [41]. *Drosophila melanogaster* gene ID and description were compiled from Flybase [64].
(XLSX)

Table S2 *A. aegypti* genes that are only methylated in the presence of *w*MelPop.
(XLSX)

Table S3 *A. aegypti* genes that are demethylated in the presence of *w*MelPop.
(XLSX)

Table S4 Transcriptional profile of head and muscle tissue of *A. aegypti* in response to *w*MelPop infection on each of two different tabs. The occurrence of false positives was corrected using the q-value [48].
(XLSX)
