# Supplementary figures and images for: Infection with a Virulent Strain of Wolbachia Disrupts Genome Wide-Patterns of Cytosine Methylation in the Mosquito Aedes aegypti
Source: PLoS One. 2013 Jun 19;8(6):e66482. doi: 10.1371/journal.pone.0066482 (PMC3686743; doi:10.1371/journal.pone.0066482)

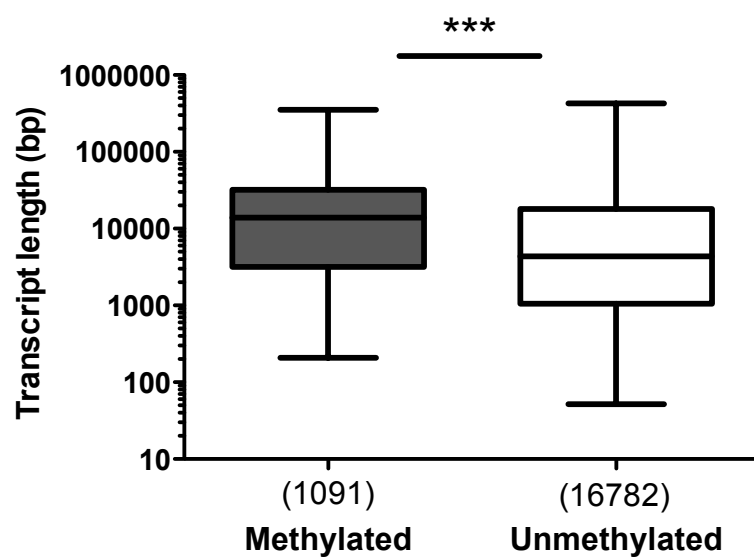

Supplement: Figure S1 — Gene region with methylation are associated with greater transcript length as compared to gene regions without methylation. Median transcript length (bp) ± interquartile range for genes whose promoters are naturally methylated or unmethylated in A. aegypti. *** P-value<0.001, n in parentheses. (PDF) [file pone.0066482.s001.pdf]

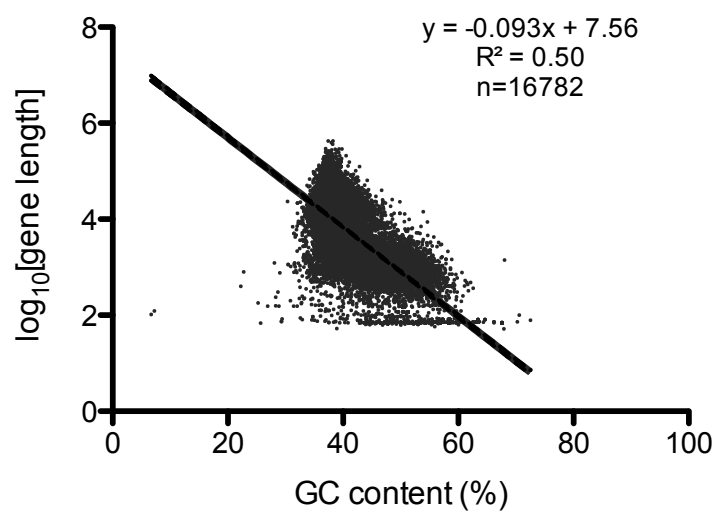

Supplement: Figure S2 — A negative correlation between GC% content of genes and gene length. (PDF) [file pone.0066482.s002.pdf]
